# Multiple interspecific hybridization and microsatellite mutations provide clonal diversity in the parthenogenetic rock lizard *Darevskia armeniaca*

**DOI:** 10.1186/s12864-018-5359-5

**Published:** 2018-12-29

**Authors:** Anastasiya E. Girnyk, Andrey A. Vergun, Seraphima K. Semyenova, Andrei S. Guliaev, Marine S. Arakelyan, Felix D. Danielyan, Irena A. Martirosyan, Robert W. Murphy, Alexey P. Ryskov

**Affiliations:** 1 Laboratory of Genome Organization, Institute of Gene Biology of the Russian Academy of Sciences, Vavilova Str., 34/5, Moscow, 119334 Russia; 2 Department of Biochemistry, Molecular biology and Genetics, Institute of biology and chemistry, Moscow State Pedagogical University, M. Pirogovskaya Str., 1/1, Moscow, 119991 Russia; 3 Faculty of Biology, Yerevan State University, 1 Alex Manoogian, 0025 Yerevan, Armenia; 4 Department of Natural History, Royal Ontario Museum, 100 Queen’s Park, Toronto, ON M5S 2C6 Canada

**Keywords:** *Darevskia*, Hybridization, Parthenogenesis, Microsatellites, SNP markers, Clones, Mutations, Clonal diversity

## Abstract

**Background:**

The parthenogenetic Caucasian rock lizard *Darevskia armeniaca*, like most other parthenogenetic vertebrate species, originated through interspecific hybridization between the closely related sexual *Darevskia mixta* and *Darevskia valentini*. *Darevskia armeniaca* was shown to consist of one widespread allozyme clone and a few rare ones, but notwithstanding the origin of clonal diversity remains unclear. We conduct genomic analysis of *D. armeniaca* and its parental sexual species using microsatellite and SNP markers to identify the origin of parthenogenetic clonal lineages.

**Results:**

Four microsatellite-containing loci were genotyped for 111 specimens of *D. armeniaca*, 17 *D. valentini*, and four *D. mixta*. For these species, a total of 47 alleles were isolated and sequenced. Analysis of the data revealed 13 genotypes or presumptive clones in parthenogenetic *D. armeniaca*, including one widespread clone, two apparently geographically restricted clones, and ten rare clones. Comparisons of genotype-specific markers in *D. armeniaca* with those of its parental species revealed three founder-events including a common and two rare clones. All other clones appeared to have originated via post-formation microsatellite mutations in the course of evolutionary history of *D. armeniaca*.

**Conclusion:**

Our new approach to microsatellite genotyping reveals allele-specific microsatellite and SNP markers for each locus studied. Interspecies comparison of these markers identifies alleles inherited by parthenospecies from parental species, and provides new information on origin and evolution of clonal diversity in *D. armeniaca*. SNP analyses reveal at least three interspecific origins of *D. armeniaca*, and microsatellite mutations in these initial clones give rise to new clones. Thus, we first establish multiple origins of *D. armeniaca.* Our study identifies the most effective molecular markers for elucidating the origins of clonal diversity in other unisexual species that arose via interspecific hybridization.

**Electronic supplementary material:**

The online version of this article (10.1186/s12864-018-5359-5) contains supplementary material, which is available to authorized users.

## Background

Naturally occurring unisexual reproduction occurs in less than 0.1% of all vertebrate species [1, 2] and true parthenogenesis has been described only in squamates [1, 3–5]. Recently, the origin and evolution of parthenogenesis, in particular in lizards, has received considerable attention [6–13]. Unisexuality usually arises through interspecific hybridization between genetically related sexual species [14, 15] and introgressive hybridization is common among species of lizards Jancuchova-Laskova et al. [7]. Regardless, hybridization between the presumed ancestral species of parthenogenetic lineages of Caucasian rock lizards (*Darevskia*) does not necessary result in parthenogenesis [16]. Among Squamata, many parthenogenetic species are triploid (e.g., some *Aspidoscelis*), however, all unisexual species of *Darevskia* (Lacertidae) are diploid. Due to their hybrid origin, these species have the genetic diversity of their parental sexual progenitors and commonly exhibit fixed heterozygosity at codominant loci [17]. Sister-chromatid pairing maintains heterozygosity, which is critical for offsetting the reduced fitness in parthenogenetic species [18].

Genetic studies of unisexual vertebrate species rely on assessments of intraspecific variability and clonal diversity. Their genetic diversity may owe to having the original clones but from different founders, post-formation mutations (especially in hypervariable microsatellite loci), and genetic recombination in rare cases of subsequent crossings [19, 20]. Range-size, the age of species and some environment factors may also affect clonal diversity [21–23].

Darevsky (1958) first discovered parthenogenesis in the family Lacertidae [24]. Later, other parthenogenetic species were described [25, 26]. *Darevskia* has at present 24 sexual and seven parthenogenetic species. [11, 14, 25]. The phylogeny of *Darevskia* reconstructed using mitochondrial DNA sequences and allozyme data [27] includes the *D. caucasica*, *D. saxicola* and *D. rudis* major groups of sexual species. The formation of parthenospecies is constrained phylogenetically [7, 27]. They arose from hybridization of females in the *D. caucasica* group and males in the *D. rudis* group. Analysis of DNA fingerprint markers has identified genetic polymorphism in all parthenogenetic species of *Darevskia* [28–32].

*Darevskia armeniaca* has a wide distribution involving central Armenia, southern Georgia, northeastern Anatolia and northwestern Azerbaijan [33–35]. They live at elevations between 800 and 2700 m [33, 36]. This species arose from the hybridization of *Darevskia mixta* (*D. caucasica* group) and *Darevskia valentini* (*D. rudis* group) [14, 15, 23, 27, 37, 38]. *Darevskia mixta*, which occurs in the eastern part of the Meskheti Range, Lesser Caucasus Mountains and on southern slops of the Greater Caucasus between the valleys of the Rioni and Khobi rivers, is endemic to Georgia. The central part of the Lesser Caucasus was the most likely area of origin for *D. mixta* [39]. In comparison, *D. valentini* occurs in eastern Anatolia, Armenia, and adjoining Georgia [15, 33] at elevations between 1900 and 3110 m. In Armenia, this species is locally abundant in montane habitats [33]. Triploid hybrids between *D. armeniaca* and sexual *D. valentini* occur in some Armenian populations [34, 40, 41]. The zone of sympatry in Kuchak has an extremely high number of hybrids [41]. The majority of them are sterile triploid females, but males with fully developed reproductive systems and presumably fertile females also exist [41]. Putative fertile triploids probably exist in *D. unisexualis*, and in some other parthenogenetic species of reptiles [41–44]. In most cases, spatial gaps separate the ranges of sexual and unisexual populations of *Darevskia* [35]. Unisexual forms have a superior competitive ability to their parental forms in a given spatio-temporal setting [36], expansion of the parthenogens could cause retreat of parental species from the contact zones, preventing further hybridization events [35].

Recently, Tarkhnishvili et al. (2017) postulated alternative hypothesis of origin of *D. armeniaca*. Apparently, it arose from backcrosses of male *D. valentini* with parthenogenetic *D. dahli* [13]. Mitochondrial DNA analysis confirmed data of Fu et al. (1999) that *D. armeniaca* and *D. dahli* descend maternally from *D. mixta* from a limited geographic area in central Georgia [45]. The majority of both parthenogens shared the same genotypes at two microsatellite loci, but they differed at the other three loci used. Therefore, they suggested that the origin of *D. armeniaca* first included hybridization between *D. mixta* and *D. portschinskii* followed by backcrosses of these parthenogens with male *D. valentini* [13]. However, new studies are needed to determine which of two or both scenarios of *D. armeniaca* origin have matter.

To test the alternative hypotheses, we examine the clonal diversity and its origin in *D. armeniaca* using 111 samples and microsatellite genotyping and single nucleotide polymorphisms (SNPs) located outside of each microsatellite cluster [46, 47]. The SNPs data yield direct information about interspecific hybridization founder events, and microsatellite variability provides information about possible mutations in the initial clones. Further, analyses use partial sequences from the mitochondrial gene encoding cytochrome *b* (*MT-CYTB*) from 14 Armenian populations of *D. armeniaca* plus the parental species.

## Methods

**Fig. 1:**
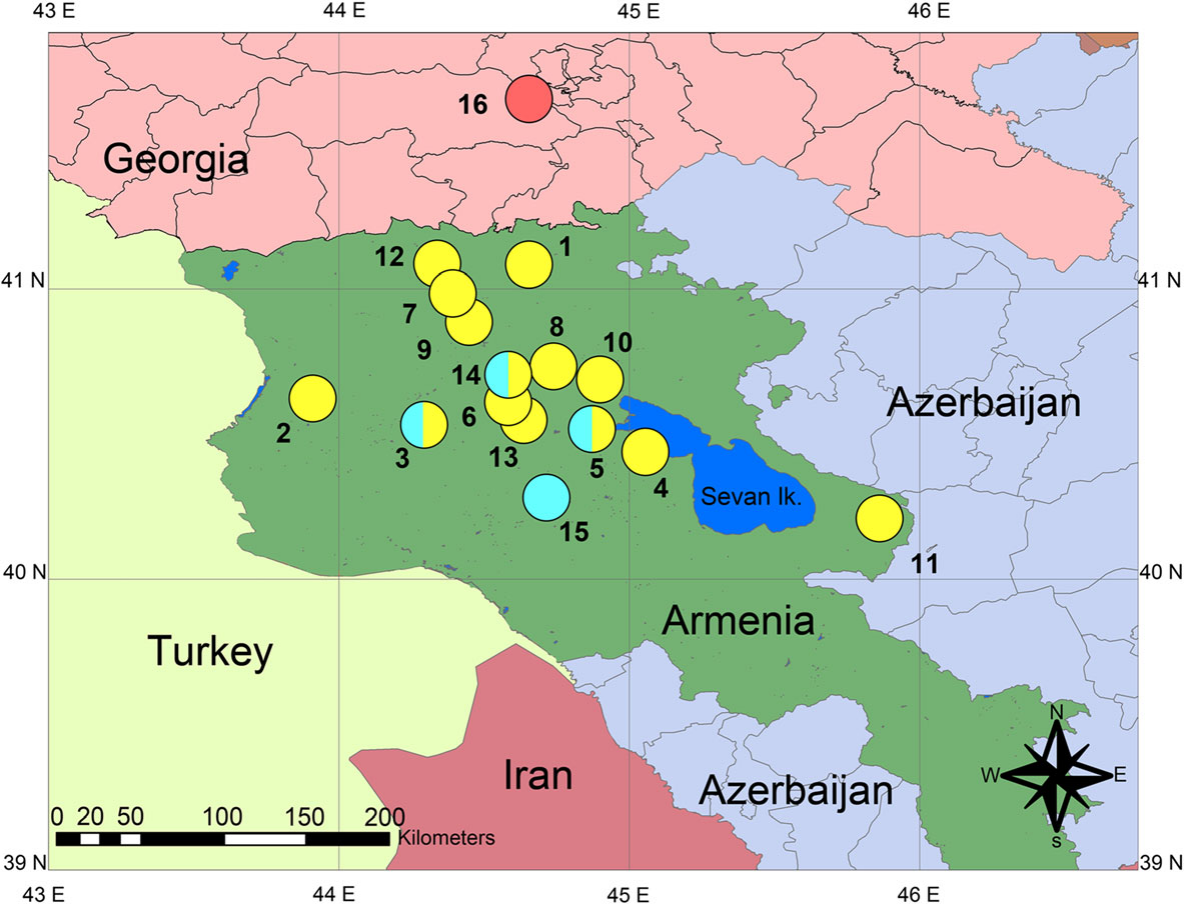
Collection localities of parthenogenetic lizards *Darevskia armeniaca* and their parental species *D. valentini* and *D. mixta*. Sampling localities are indicated by the following colors: *D. armeniaca* – yellow; *D. valentini –* blue; *D. mixta –* red. Numbers indicate populations: 1 – Dsegh (41°04′50.8″N 44°39′27.1″E); 2 - Harich (40°38′25.9″N 43°54′14.4″E); 3 - Kuchak (40°31′ 49.81″N 44°17′3.43″E); 4 - Sevan (40°28′02.4″N 45°03′43.5″E); 5 - Lchashen (40°30′45.92″N 44°54′3.22″E); 6 - Meghradzor (40°36′45.1″N 44°36′23.5″E); 7 - Medved-gora (vicinity of Stepanavan) (40°58′45.8″N 44°24′32.7″E); 8 - Dilijan (Papanino) (40°42′27.76″N 44°45′43.89″E); 9 - Pushkin Pass (40°54′42.1″N 44°25′55.6″E); 10 - Dilijan-Semyonovka Pass (40°39′52.6″N 44°53′24.4″E); 11 - Sotk (40°12′43.8″N 45°52′42.6″E); 12 - Stepanavan (41°03′21.8″N 44°21′33.5″E); 13 - Artavaz (Hankavan) (40°37′20.2″N 44°34′51.4″E); 14 - Tezh (Pambak Ridge) (40°42′8.08″N 44°36′30.80″E); 15 - Hatis (Geghama Mountains) (40°18′14.91″N 44°43′40.71″E); 16 - Akhaldaba (Georgia) (41°41′3.840″N 44°39′29.880″E). The map was created in the licensed version ArcGIS Desktop 10.4.1 by the authors (http://desktop.arcgis.com)

DNA samples of *D. armeniaca* (*n* = 111) from 14 populations, *D. valentini* (*n* = 17) from four populations in Armenia, and *D. mixta* (*n* = 4) from one population in Georgia were analyzed (Table 1). Sample localities were shown in Fig. 1.

**Table 1:** Species and population samples used in this study

Species	Populations	Number of individuals in populations	Total number of species individuals
*D. armeniaca*	Dsegh	3	111
	Harich	18	
	Kuchak	7	
	Sevan	1	
	Lchashen	1	
	Meghradzor	9	
	Medved-gora (vicinity of Stepanavan)	12	
	Dilijan (Papanino)	4	
	Pushkin Pass	7	
	Dilijan-Semyonovka Pass	8	
	Sotk	3	
	Stepanavan	9	
	Artavaz (Hankavan)	21	
	Tezh (Pambak Ridge)	8	
*D. valentini*	Hatis (Geghama Mountains)	4	17
	Kuchak	2	
	Lchashen	5	
	Tezh (Pambak Ridge)	6	
*D. mixta*	Akhaldaba (Georgia)	4	4

All DNAs were isolated from blood samples of lizards. Lizards were captured by Danielyan F.D. and Arakelyan M.S. 15–20 years ago with a noose. These species are not protected by CITES. The study was approved by the Ethics Committee of Moscow State University (Permit Number: 24–01) and was carried out in strict accordance with their ethical principles and scientific standards. Blood samples were taken from the tail veins of lizards under chloroform anesthesia, and then these lizards were released. DNA was isolated from lizard blood by using the standard phenol–chloroform extraction method with proteinase K, and resuspended in TE buffer, pH 8.0.

Four loci, Du215, Du281, Du323, and Du47G were PCR-amplified using previously described primer pairs [46, 48, 49]. Data on genetic variation of these loci in *D. armeniaca* and on allelic variation of the homologous loci in *D. valentini* and *D. mixta* [49] were shown in Additional file 1: Table S1. PCR was performed on 50 ng of DNA in a total volume of 20 μl using a GenePak PCR Core Kit (Isogene) and 1 μM of each primer. The reaction conditions were as follows: one cycle of 3 min at 94 °C; 30 cycles of 1 min at 94 °C; 40s at the annealing temperature (58 °C for Du215, 50 °C for Du281, 52 °C for Du323, and 54 °C for Du47G); and 40s at 72 °C followed by one cycle of 5 min at 72 °C. PCR products (7 μl) were loaded onto an 8% non-denatured polyacrylamide gel (to separate allelic variants for each locus) and run for 12 h at 60 V. A 100 bp ladder (Fermentas) was used as a size marker. The amplified products were visualized by staining DNA in the gel with ethidium bromide. Well-resolved individual PCR products, which corresponded to the two individual alleles of the locus, were excised from the gel, purified by ethanol precipitation, and sequenced directly in both directions using a chain termination reaction with an ABI PRISM BigDye Terminator v.3.1 on an Applied Biosystems 3730 DNA analyzer. Allelic identity was checked and confirmed via the comparison of sequences obtained independently. All unique de novo sequences were deposited in GenBank (GU972533-GU972535; HM070259-HM070264; HM013992-HM013994; KT070998-KT071004; GU972551; GU972553; HM013997; KM573717-KM573727; MH187988-MH187999). Sometimes for genotypes 9–13, which were represented by only one individual, the PCR products of the Du47G locus were excised from the polyacrylamide gel, purified and cloned into pMos blue vectors according to standard procedures (pMos blueBlunt ended Cloning kit RPN 5110, Amersham Biosciences). The clones were amplified in MOSBlue competent cells grown at 37 °C, and sequenced as described previously [50].

Diversity parameters for each locus were estimated only for *D. armeniaca*, because sample sizes of the parental sexual species were insufficient. The number of alleles, allelic richness (Rs), as a measure of allele counts adjusted for sample size, and expected heterozygosity, as a measure of gene diversity, were calculated per locus and per population by using R package Poppr v.2.5.0 [51], GenePop v.4.2, and Web-version of POPTREEW [52].

A statistical parsimony haplotype network was used to visualize variation. It was calculated using TCS software v.1.21, with gaps being considered as a second state [53]. This approach has been used at the population-level for comparing mitochondrial SNPs, which have linear arrangements [54]. Clonality in parthenogenetic lizards resulted in homologous alleles having linear arrangements with little or no recombination. This served to link the parthenogenetic genotypes with mutations (repeat number changes). Tarkhnishvili et al. (2017) used a similar approach for linking microsatellite genotypes of *D. dahli* and *D. armeniaca* from Georgia, Armenia and Turkey, but with Network v.5.0 [13, 55]. The method of coding genotypes was described previously [46].

A 320 bp fragment of *MT-CYTB* was amplified and sequenced for 30 specimens of *D. armeniaca* (2–3 individuals from each population) including those with a distinct microsatellite genotype, and four specimens of *D. mixta* from one population*.* The primers used were L14841: 5′-CCATCCAACATCTCAGCATGATGAAA-3′ and H15149: 5′-GCCCCTCAGAATGATATTTGTCCTCA-3′ [56], as described for these taxa in [27]. PCR analysis was performed as described in [13]. The amplicons were sequenced on an automated sequencer (Applied Biosystems 3730 DNA analyzer). Sequence alignment was performed with BioEdit v.7.0 [57].

## Results

All 111 specimens of *D. armeniaca*, including 10 individuals with coloration difference [37] from Artavaz (Hankavan), 17 specimens of *D. valentini*, and four *D. mixta* were analyzed successfully using locus-specific PCR and DNA sequencing of PCR amplificants. The 320 bp fragment of *MT-CYTB* did not vary among *D. armeniaca* and *D. mixta*; all sequences assigned to haplotype A of *D. mixta* [45].

All but four individuals of *D. armeniaca* (from Dsegh, Dilijan-Semyonovka Pass and Stepanavan) were heterozygous at microsatellite loci used; the alleles differed from each other in length and structure, and in single nucleotide variations (SNVs) in fixed positions of the flanking allelic regions (Additional file 1: Table S1), which was similar to previous reports for *D. dahli*, *D. unisexualis*, and *D. rostombekowi* [32, 46–48]. Locus Du323, also had a unique (AC)_n_ microsatellite cluster that differentiated parental alleles.

In *D. armeniaca*, Du215 and Du323 had three alleles, Du281 had four, and Du47G had seven (Additional file 1: Table S1). Allelic variants formed distinct groups according to the fixed SNVs, which in hybrid genomes resulted from the different parental genomes. They corresponded to parent-specific markers such that unique clonal SNVs likely reflected independent founder events. SNVs in alleles 1 and 2 of Du215 formed the set TGC and allele 3 of Du215 formed the set ACT. Similarly, SNVs in alleles of Du281 had T and C variants, and SNVs in alleles of Du323 had CT and AC variants. The seven alleles of Du47G formed three sets of SNVs: TAGT, TTCA, and AAGA. Each sets associated with specifically organized microsatellite clusters. Thus, together the sets of SNVs and microsatellite clusters differentiated distinct genotypes inherited by parthenospecies from parental species.

Analysis of the parental species showed homozygosity for Du215 and Du323 in *D. mixta* (*n* = 4), and Du281 and Du47G had two alleles each. In *D. valentini* (*n* = 17), Du215 was homozygous, Du281 had five, Du323 had six, and Du47G had 10 alleles (Additional file 1: Table S1). All parental alleles contained microsatellite clusters and variable nucleotides (SNVs) at fixed positions in neighboring regions which differentiated *D. mixta* and *D. valentini*.

**Fig. 2:**
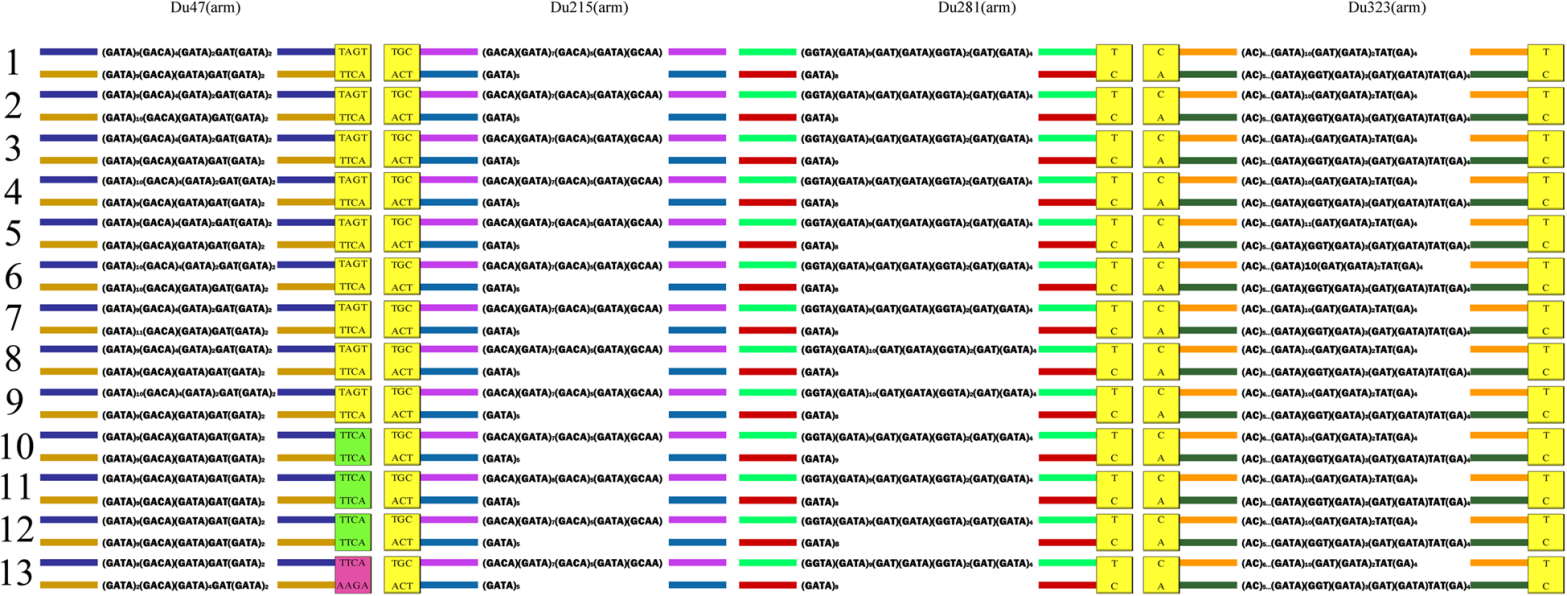
Schematic representation of thirteen genotypes formed by allelic combinations of microsatellite loci Du215, Du281, Du323, and Du47G in 111 individuals of *D. armeniaca*. Parent-specific SNV markers are shown in yellow squares. Variable microsatellite clusters are shown in each of two alleles

Microsatellite motifs and/or the sets of SNVs discerned parental alleles*.* Most alleles of Du215, Du281, Du323, and Du47G in *D. armeniaca* occurred in the parents. Alleles Du215(arm)1 and 2 coincided with allele Du215(mix)1, and allele Du215(arm)3 coincided with Du215(val)1. Further, alleles Du281(arm)1 and 2 coincided with Du281(mix)1 and 2, and alleles Du281(arm)3 and 4 coincided with Du281(val)1–5. Alleles Du323(arm)1 and 2 with the specific (AC)_6_ microsatellite cluster occurred among Du323 alleles from *D. valentini*, and allele Du323(arm)3 with specific (AC)_5_ microsatellite cluster coincided with Du323(mix)1. Finally alleles, Du47G(arm)3–6 corresponded to alleles of Du47G in *D. valentini*, and Du47G(arm)7 coincided with Du47G(mix)2. Due to the high mutation rate of microsatellite DNAs [50], in some cases we did not find direct correlation between distinct alleles of *D. armeniaca* and its parents. Parental alleles with the SNV TAGT, which was specific for Du47G(arm)1 and 2, was not found probably due to either genome divergence through genetic recombination or mutation in *D. armeniaca*, or insufficient parental sampling. Allelic combinations of four loci identified genotypic diversity in *D. armeniaca* (Fig. 2).

Analyses resolved 13 genotypes that differed in their frequencies and distribution (Table 2). Widespread genotype 1 occurred in 61 individuals (54.9% of the total cohort). Genotype 4 (*n* = 10; 9%) and genotype 6 (*n* = 5; 4.5%) were found in four and three populations, respectively. All other genotypes (2, 3, 5, 7–13) occurred in one or two populations (*n* = 35; 32.5%). Although relatively uncommon, genotypes 2–5 made up each the majority of individuals in some populations: genotype 2 at Harich (12 of 18 specimens), genotype 3 at Kuchak (5 of 8), genotype 4 at Pushkin Pass (5 of 7), and genotype 5 at Lchashen (7 of 9) (Table 2). All 10 color-variant individuals from Artavaz (Hankavan) had genotype 1.

**Table 2:** Sample size, genotype composition, diversity and distribution in the populations of *D. armeniaca*

Genotype number	Genotype composition (see, Fig. 2)	Population														
		1	2	3	4	5	6	7	8	9	10	11	12	13	14	Number of individuals with definite genotype (genotype frequencies)
1	Du215(2 + 3) + Du281(2 + 4) + Du323(2 + 3) + Du47G(2 + 5)			6	1	1	2	10	3	2	1	1	5	21	8	61 (0,549)
2	Du215(2 + 3) + Du281(2 + 4) + Du323(2 + 3) + Du47G(2 + 4)		12										1			13 (0,117)
3	Du215(2 + 3) + Du281(2 + 3) + Du323(2 + 3) + Du47G(2 + 5)										5					5 (0,045)
4	Du215(2 + 3) + Du281(2 + 4) + Du323(2 + 3) + Du47G(1 + 5)	2	1					2		5						10 (0,090)
5	Du215(2 + 3) + Du281(2 + 4) + Du323(1 + 3) + Du47G(2 + 5)						7									7 (0,063)
6	Du215(2 + 3) + Du281(2 + 4) + Du323(2 + 3) + Du47G(1 + 4)		2									2	1			5 (0,045)
7	Du215(2 + 3) + Du281(2 + 4) + Du323(2 + 3) + Du47G(2 + 3)		2						1							3 (0,027)
8	Du215(2 + 3) + Du281(1 + 4) + Du323(2 + 3) + Du47G(2 + 5)			1												1 (0,009)
9	Du215(2 + 3) + Du281(1 + 4) + Du323(2 + 3) + Du47G(1 + 5)												1			1 (0,009)
10	Du215(2 + 3) + Du281(2 + 3) + Du323(2 + 3) + Du47G(5 + 5)										2					2 (0,018)
11	Du215(1 + 3) + Du281(2 + 4) + Du323(2 + 3) + Du47G(5 + 5)	1														1 (0,009)
12	Du215(2 + 3) + Du281(2 + 4) + Du323(2 + 3) + Du47G(5 + 5)												1			1 (0,009)
13	Du215(2 + 3) + Du281(2 + 4) + Du323(2 + 3) + Du47G(6 + 7)		1													1 (0,009)
Total number of individuals		3	18	7	1	1	9	12	4	7	8	3	9	21	8	111
Genotype diversity (%)		2 (66,7)	5 (27,8)	2 (28,6)	1 (0)	1 (0)	2 (22,2)	2 (16,7)	2 (50)	2 (28,6)	3 (37,5)	2 (66,7)	5 (55,5)	1 (0)	1 (0)	13

Genotypic diversity of *D. armeniaca* varied from 0 to 66.7% (Table 2). The highest level of genotypic diversity was observed at Dsegh and Sotk, which had two genotypes in three individuals. Five genotypes, one common and four rare, occurred in the nine individuals from Stepanavan. The lowest levels of genotypic diversity were observed at Sevan, Lchashen, Artavaz (Hankavan), and Tezh (Pambak Ridge), which had genotype 1 only.

Population genetic indices for four loci and genotypes 1–9 were given in Table 3. Five individuals with unique genotypes 10–13, and populations at Sevan and Lchashen, which were represented by one individual each, were excluded from the analysis. The estimates of expected heterozygosity varied from 0.51 to 0.67 (average 0.55–0.63 depending on locus) whereas observed heterozygosity did not vary among loci and populations (Table 3). The number of alleles varied from 2 to 5 (average, 2.00–2.83 depending on locus). Values of allelic richness ranged from 1.89 to 3.20 (average, 1.94–2.24 depending on locus). The highest values of heterozygosity occurred at Dsegh (loci Du215, Du281, Du323) and Sotk (locus Du47G). The highest values of allelic richness occurred at Dsegh (loci Du215 and Du281), Sotk (loci Du215, Du281, and Du47G) and Medved-gora (vicinity of Stepanavan) (locus Du323), while the highest values of allelic number varied from 2 to 5 depending on the locus in the populations studied.

**Table 3:** The population indices of gene diversity for four studied loci in twelve sampled populations of *D. armeniaca*

Locus	Population	Alelle (N)	R_S_	H_E_	H_O_
Du215	Dsegh	2	2.00	0.67	1
	Harich	2	1.89	0.52	1
	Kuchak	2	1.93	0.54	1
	Meghradzor	2	1.90	0.53	1
	Medved-gora (vicinity of Stepanavan)	2	1.92	0.52	1
	Dilijan (Papanino)	2	1.97	0.57	1
	Pushkin Pass	2	1.93	0.54	1
	Dilijan-Semyonovka Pass	2	1.93	0.54	1
	Sotk	2	2.00	0.60	1
	Stepanavan	2	1.92	0.53	1
	Artavaz (Hankavan)	2	1.89	0.51	1
	Tezh (Pambak Ridge)	2	1.92	0.53	1
	Total	2	2.00	0.50	1
	Mean ± SE	2 ± 0.00	1.94 ± 0.01	0.55 ± 0.01	1 ± 0.00
Du281	Dsegh	2	2.00	0.67	1
	Harich	2	1.89	0.52	1
	Kuchak	3	2.18	0.60	1
	Meghradzor	2	1.91	0.53	1
	Medved-gora (vicinity of Stepanavan)	2	1.92	0.52	1
	Dilijan (Papanino)	2	1.97	0.57	1
	Pushkin Pass	2	1.93	0.54	1
	Dilijan-Semyonovka Pass	3	2.18	0.60	1
	Sotk	2	2.00	0.60	1
	Stepanavan	3	2.14	0.59	1
	Artavaz (Hankavan)	2	1.89	0.51	1
	Tezh (Pambak Ridge)	2	1.92	0.53	1
	Total	4	4.00	0.54	1
	Mean ± SE	2.25 ± 0.13	2.00 ± 0.03	0.56 ± 0.01	1 ± 0.00
Du323	Dsegh	2	2.00	0.67	1
	Harich	2	1.89	0.52	1
	Kuchak	2	1.93	0.54	1
	Meghradzor	3	1.91	0.62	1
	Medved-gora (vicinity of Stepanavan)	2	2.26	0.52	1
	Dilijan (Papanino)	2	1.97	0.57	1
	Pushkin Pass	2	1.93	0.54	1
	Dilijan-Semyonovka Pass	2	1.93	0.54	1
	Sotk	2	2.00	0.60	1
	Stepanavan	2	1.92	0.53	1
	Artavaz (Hankavan)	2	1.89	0.51	1
	Tezh (Pambak Ridge)	2	1.92	0.53	1
	Total	3	3.00	0.53	1
	Mean ± SE	2.08 ± 0.08	1.96 ± 0.03	0.56 ± 0.01	1 ± 0.00
Du47G	Dsegh	2	2.00	0.67	1
	Harich	5	2.45	0.67	1
	Kuchak	2	1.93	0.54	1
	Meghradzor	2	2.17	0.53	1
	Medved-gora (vicinity of Stepanavan)	3	1.92	0.59	1
	Dilijan (Papanino)	3	2.41	0.68	1
	Pushkin Pass	3	2.34	0.65	1
	Dilijan-Semyonovka Pass	2	1.92	0.53	0.86
	Sotk	4	3.20	0.87	1
	Stepanavan	4	2.67	0.73	1
	Artavaz (Hankavan)	2	1.89	0.51	1
	Tezh (Pambak Ridge)	2	1.92	0.53	1
	Total	5	5.00	0.65	1
	Mean ± SE	2.83 ± 0.30	2.24 ± 0.12	0.63 ± 0.03	0.93 ± 0.07

*N* Number of alleles, *R*_*S*_ Allelic richness, H_E_ Expected heterozygosity, *H*_*O*_ Observed heterozygosity

Population genetic indices for four loci and four populations (17 individuals) were given in the Additional file 2: Table S2. The estimates of expected heterozygosity varied from 0.50 to 0.89 (average 0.52–0.84 depending on locus), whereas observed heterozygosity from 1 for locus Du215 (all populations) to 0.33–0.94 for other loci. The number of alleles varied from 2 to 6 (average, 2–5 depending on locus). Values of allelic richness ranged from 1.79 to 3.43 (average, 1.96–3.36 depending on locus). The highest value of allelic richness occurred in the population at Lchashen (loci Du47G, Du323, Du215). The highest values of expected heterozygosity occurred in Adis (locus Du47G). These data demonstrated that higher genetic variability in populations of *D. valentini* rather than populations of *D. armeniaca*. Linkage disequilibrium analysis (Additional file 3: Table S3) suggested that all populations except Lshachen had no association between loci. The population at Lshachen appeared to have linked loci probably because of either a recent bottleneck or association of microsatellite markers with unknown loci under selection.

Combinations of parent-specific SNVs and those of *D. armeniaca* differentiated between single or multiple interspecies hybridization event(s). The structural composition of the 13 genotypes (Additional file 1: Table S1) were shown schematically in Fig. 2. Genotypes 1–9 matched all parent-specific SNV combinations TAGT/TTCA (Du47G), TGC/ACT (Du215), T/C (Du281), and CT/AC (Du323). This did not reject the hypothesis of a common origin from a single hybridization event. However, the analysis did not rule out the possibility of independent crossings of the parental individuals because they differed from each other by microsatellite sequences only at loci Du47G, Du281, and Du323. Some of the rarer genotypes may have arisen via post-formation microsatellite mutation.

Genotypes 10–12, which occurred in four individuals from three populations, matched parent-specific SNV combinations at all loci, but they differed from genotypes 1–9 by the parent-specific SNV combination TTCA/TTCA for locus Du47G. These four specimens were uniquely homozygous at Du47G and differed from each other only by microsatellite sequences at Du215 and Du281. Therefore, this rejects the hypothesis of a single origin of *D. armeniaca*, and their variation likely arose through microsatellite mutations. Further, rare genotype 13 (*n* = 1) differed from all other genotypes by the SNV combination TTCA/AAGA at locus Du47G. This also rejected the null hypothesis. Consequently, analyses pointed to at least three independent hybridization events in the genesis of the 13 genotypes of *D. armeniaca*.

The TCS network displayed the geographical distribution of genotypes 1–9 (Additional file 4: Figure S1). The network displayed a star-like appearance that was typical of mutations deriving from a central, ancestral genotype.

## Discussion

In their review of allozyme variation in parthenogenetic lizards, Parker et al. (1989) proposed that species having a single origin will usually have a widespread numerous clone along with a few rare clones, where species with multiple hybrid origins are highly variable with random allele combinations [22]. In some cases, high diversity in allozymes but low variation in mitochondrial DNA suggests multiple origins from a geographically restricted sample of females [22, 58, 59]. Add to this, low diversity in allozymes and mitochondrial DNA suggests restricted origins, both geographically and numerically [60, 61]. In a laboratory creation of hybridogenetic *Poeciliopsis* lineages, relatively low clonal diversity resulted from selection of the most-fit clones from a broad spectrum of genotypes [2, 16].

The clonal diversity of parthenogenetic species was detailed using allozyme and mitochondrial DNA analysis [16, 23, 27, 38, 45, 62–65]. Allozyme analyses resolved several clones in all species except for *D. rostombekowi*), and all consist in one major widespread clone and a few rare clones. Some peculiarities in allozyme clone patterns were found for *D. armeniaca* [38, 66].

*Darevskia armeniaca* has a karyotype of 2n = 38 [67] characterized by fixed heterozygosity of allozyme loci [38, 66] and exhibits no [45] or low variability of mitochondrial DNA inherited from *D. mixta* [27]. Using five loci, Uzzell and Darevsky (1975) found one clone in 11 specimens of *D. armeniaca* from two populations from Armenia [14]. MacCulloch et al. (1995) examined 35 loci and 75 specimens from seven Armenian populations and found one widespread clone with two rare clones [38]. One of the rare clones occurred in 19 out of 27 individuals at Dilijan (Papanino) and the other one the only clone in Kuchak (*n* = 2). Fu et al. (2000) used 35 allozyme loci and 117 specimens, including some lizards with the color variation described by Darevsky (1992) and Danielyan (1999) as being four clones, one being common [37, 66, 68]. One rare allozyme clone dominated in two populations and a rare color-variant clone at Ankavan (n = 2) differed at two loci [66]. This is unlike *D. dahli*, whose morphological difference did not correspond with either allozyme or microsatellite markers [16, 46].

MacCulloch et al. (1995) attributed the clonal variation that associated with morphological data in *D. armeniaca* to mutations [37, 38]. They argued that (1) some rare clones had alleles not detected in the parental species; (2) the pattern of clonal variation in *D. armeniaca* was typical of parthenogenetic lizards of single hybridization origin such as in teiid *Aspidocelis* [22]; and (3) overall variation in *D. armeniaca* was less than that found in species of multiple hybrid origin, such as parthenogenetic gecko *Heteronatia binoei* [19]. Alternatively, Fu et al. (2000) suggested that although mutation was a possible explanation of the origin of the clonal variation in *D. armeniaca*, but multiple origins was equally likely [66]. The rare clones in the Kuchak and Dilijan (Papanino) may have owed to an independent origins because of dominance of rare alleles and (2) the peripheral distribution to the common clone suggested multiple origin [22]. Further, the young age of *D. armeniaca* implied from low mtDNA variation and substantial allozyme variation favored the multiple-origin scenario over a rapid accumulation of mutations [66].

Our analyses indicate that at least three interspecific hybridizations were involved in the genesis of *D. armeniaca* and microsatellite mutations followed these. A previous allozyme analysis reported four clones with the rare ones occurring in restricted populations [65]. This allozyme pattern generally followed Parker et al.’s (1989) model [22], which is consistent with single hybridization origin. Nevertheless, Fu et al. (2000) noted that multiple origins could also explain the clonal diversity [66]*.*

Our modified microsatellite genotyping [46] detects both microsatellite and SNVs variability. The method reveals 13 genotypes in 111 individuals using four microsatellite loci. Previously, the approach revealed a high level of clonal diversity (11 clones) in *D. dahli* [46] and rejected the hypothesis of monoclonality of *D. rostombekowi* [47], and our analyses also detect greater variation in *D. armeniaca* than did allozymes. Thus, this method is a more precise measure of assessing genetic variability compared with allozymes.

Previously, the two color-variant individuals of *D. armeniaca* from Ankavan were investigated using allozymes [65]. These specimens differed from the common allozyme clone forming separate clones. However, our data failed to distinguish 10 specimens of *D. armeniaca* with differences in coloration; they all have the most abundant and widespread genotype 1. The color-variants consist of about half of the samples from Artavaz (Hankavan). Similarly, our microsatellite genotyping data [46], as well as allozyme [16] and mtDNA [23] studies, failed to distinguish the color-varieties of parthenogenetic *D. dahli*.

The pattern of distribution of the clones rejects the hypothesis of a single origin for *D. armeniaca*. All individuals with genotypes 1–9 exhibit an identical combination of parent-specific SNVs, suggesting their common origin via one hybridization event. Given that *D. mixta* and *D. valentini* are the parental species for *D. armeniaca* [14, 23, 37, 38, 66], the identical SNVs but different microsatellite markers indicates that the hybridization event involved one population. The five individuals with genotypes 10–12 also exhibit an identical combination of parent-specific SNVs, suggesting their common origin, but one of that differs from individuals with genotypes 1–9. Clonal diversity in both groups owes to microsatellite unstable (GATA)_n_ mutations only. Such mutations in parthenogenetic *D. unisexualis* occur in one generation via deletion or insertion of a single repeat at one or at both alleles of the locus [50]. Finally, the individual from Harich has genotype 13 and a unique combination of parent-specific SNVs at locus Du47G. Allele Du47G(arm)7 was inherited by *D. armeniaca* from *D. mixta* (Additional file 1:Table S1). Therefore, this individual may represent a third hybridization event.

The rather high clonal diversity in *D armeniaca* likely owes to its multiple hybrid origin and subsequent microsatellite mutations. Similarly, most of the observed clonal diversity within parthenogenetic *D. dahli* in 9 out of 11 detected genotypes owe to microsatellite mutations within the common clone, and two out of 111 individuals were suggested to be members of independent hybridization events [46]. Analyses of parthenogenetic *D. rostombekowi* [47] revealed one common and four rare clones, each represented by one or several individuals from one or two populations. The results were consistent with single hybridization origin of *D. rostombekowi*, with clonal diversity arising via post-formation microsatellite mutations [47].

Tarkhnishvili et al. (2017) hypothesized that *D. armeniaca* originated from a series of crosses between *D. valentini* and parthenogenetic *D. dahli*, rather than between *D. valentini* and *D. mixta* [13]. Their microsatellite genotyping of Du215, Du281, Du481, Du323, and Du47G revealed identical genotypes for *D. dahli* and *D. armeniaca* at Du323 and Du47, but not at the other three loci. They suggested that backcrosses and mutations best explained the genetic and clonal diversity than a multiclonal origin. However, species of *Darevskia* exhibit rather high levels of genomic similarity and very similar microsatellite alleles. They assumed alleles of equal length had identical nucleotide sequences. Given our discoveries, more detailed molecular data on the structure of the alleles and genotypes is needed to support their hypothesis. Our discovery of multiple origins provides a more parsimonious hypothesis than the complex hypothesis of backcrossing. Further, projections on the historical distribution of sexual and parthenogenetic species during glacial waves is necessary to document the possibility of hybrids between *D. dahli* and *D. valentini*, as well as possible hybridizations between *D. mixta* and *D. portschinskii* that might be according to [13] the first stage in origin of *D. armeniaca.*

Parker et al. (1989) proposed that unisexual species originating through a single hybridization event will exhibit a common clone with a few rare clones [22]. Accordingly, among clones 1–9, genotype 1 might be ancestral (Table 2). The TCS network (Additional file 4: Figure S1) has a star-like structure with common genotype 1 occupying the central location and the others differing by from one to two mutational events. This star-like structure is consistent with a recent origin and diversification of clones*.* The same inference is not possible for genotypes 10–12 because none of them is numerous and widespread. Dsegh and Harich do not appear to have genotype 1. However, genotype 11 at Dsegh and genotype 13 at Harich arose independently from genotype 1. Genotype 4 at Dsegh and Harich, and genotypes 2, 6 and 7 at Harich appear to be variants of genotype 1. If so, then their occurrence in these localities must owe to the dispersal of lizards from other regions.

MacCulloch et al. (1995) and Fu et al. (2000) observed some peculiarities in allozyme-clone variation [62, 65]. Among their five populations of *D. armeniaca*, rare clones made up the majority of the individuals in Kuchak and Dilijan (Papanino), although the common clone covered most of the species’ distribution. This contributed to the suggestion that numerous rare clones might indicate independent hybrid origins [65]. However, our parent-specific SNVs for these clones are identical to those of genotype 1.

The distribution of microsatellite clones in *D. armeniaca* is not exceptional. Among 11 detected clones in the parthenospecies *D. dahli* [46], two were numerous and widespread geographically and all others were rare, although one rare clone in “Dendropark” (near Stepanavan) occurred in 6 out of 9 individuals. Similar analysis revealed one common and four rare clones in *D. rostombekowi*, and one of the rare clones occurred in all 8 individuals at Tsovak [47]. Thus, in all three parthenogenetic species investigated, the numerous rare clones most likely arose via post-formation microsatellite mutations of the common clone, and not through independent interspecific hybridizations. Ecological differences among the habitats of various populations, and the relative fitness of clonal lineages of *D. armeniaca* and other parthenogenetic *Darevskia* may explain the differences in the distribution and existence of successful rare clones.

In summary, the analyses suggest that the clonal diversity in *D. armeniaca* derives from three interspecific hybridizations and subsequent microsatellite mutations. Future studies may identify the role mutations play in altering of the initial clones. The methodological approach, which is based on the detection of parent-specific microsatellite and SNV markers, can elucidate the origin of genetic and clonal diversity in other unisexual species that arose via interspecific hybridization.

## Conclusions

Our interspecific genomic analysis used microsatellites and single nucleotide polymorphisms (SNP). A comparison of these markers between parthenogenetic *D. armeniaca* and its parental sexual species *D. valentini* and *D. mixta* reveals 13 genotypes or presumptive clones. Clonal diversity in *D. armeniaca* appears to result from at least three independent interspecific hybridization events. All other clones of *D. armeniaca* appear to have derived from subsequent microsatellite mutations. This methodological approach, which is based on the detection of parent-specific microsatellite and SNP markers, can be applied for study clonal diversity in other unisexual species that arose via interspecific hybridization.

## Additional files

Additional file 1: **Table S1.** Allelic variations of microsatellite containing loci in the lizard species *D. armeniaca, D. valentini,* and *D. mixta*. (PDF 132 kb)

Additional file 2: **Table S2.** The population indices of gene diversity for four studied loci in twelve sampled populations of *D. valentini. (PDF 99 kb)*

Additional file 3: **Table S3.** Indices of association in four populations of *D. valentini. (PDF 347 kb)*

Additional file 4: **Figure S1.** Schematic representation of the TCS network that reflects distribution of genotypes 1–9 in *D. armeniaca.* Concatenated sequences of *D. armeniaca* genotypes were analyzed using TCS software v.1.21. Genotypes 10–13 are plotted separately. Population distribution of the genotypes is shown by different colors. Numbers indicate the number of individuals in populations. The black circles show, unsampled, but computer-predicted genotypes. (TIF 802 kb)
