# Supplementary material for: Multiple interspecific hybridization and microsatellite mutations provide clonal diversity in the parthenogenetic rock lizard Darevskia armeniaca
Source: BMC Genomics. 2018 Dec 29;19:979. doi: 10.1186/s12864-018-5359-5 (PMC6311022; doi:10.1186/s12864-018-5359-5)
Supplement: Supplementary file 1 — Table S1. Allelic variations of microsatellite containing loci in the lizard species D. armeniaca, D. valentini, and D. mixta. (PDF 132 kb) [file 12864_2018_5359_MOESM1_ESM.pdf]

Table S1 Allelic variations of microsatellite containing loci in the lizard species *D. armeniaca*, *D. valentini*, and *D. mixta*

Allelic variant	Size (bp)	Structure of microsatellite cluster	SNV (nucleotide position, N)
**Du215(arm)1**	236	5’ (GACA)(GATA)$_{8}$(GACA)$_{5}$(GATA)(GCAA) 3’	T (–61), G (–41), C (–22)
**Du215(arm)2**	232	5’ (GACA)(GATA)$_{7}$(GACA)$_{5}$(GATA)(GCAA) 3’	T (–61), G (–41), C (–22)
**Du215(arm)3**	192	5’ (GATA)$_{5}$ 3’	A (–61), C (–41), T (–22)
Du215(mix)1	232	5’ (GACA)(GATA)$_{7}$(GACA)$_{5}$(GATA)(GCAA) 3’	T (–61), G (–41), C (–22)
Du215(val)1	192	5’ (GATA)$_{5}$ 3’	A (–61), C (–41), T (–22)
**Du281(arm)1**	229	5’ (GGTA)(GATA)$_{10}$(GAT)(GATA)(GGTA)$_{2}$(GAT)(GATA)$_{4}$ 3’	T (+15)
**Du281(arm)2**	225	5’ (GGTA)(GATA)$_{9}$(GAT)(GATA)(GGTA)$_{2}$(GAT)(GATA)$_{4}$ 3’	T (+15)
**Du281(arm)3**	187	5’(GATA)$_{9}$ 3’	C (+15)
**Du281(arm)4**	183	5’ (GATA)$_{8}$ 3’	C (+15)
Du281(mix)1	225	5’ (GGTA)(GATA)$_{9}$(GAT)(GATA)(GGTA)$_{2}$(GAT)(GATA)$_{4}$ 3’	T (+15)
Du281(mix)2	221	5’ (GGTA)(GATA)$_{8}$(GAT)(GATA)(GGTA)$_{2}$(GAT)(GATA)$_{4}$ 3’	T (+15)
Du281(val)1	199	5’ (GATA)$_{12}$ 3’	C (+15)
Du281(val)2	195	5’ (GATA)$_{11}$ 3’	C (+15)
Du281(val)3	191	5’ (GATA)$_{10}$ 3’	C (+15)
Du281(val)4	187	5’ (GATA)$_{9}$ 3’	C (+15)
Du281(val)5	183	5’ (GATA)$_{8}$ 3’	C (+15)
**Du323(arm)1**	215	5’ (AC)$_{6}$...(GATA)$_{11}$(GAT)(GATA)$_{2}$TAT 3’	C (–23), T (+36)
**Du323(arm)2**	211	5’ (AC)$_{6}$...(GATA)$_{10}$(GAT)(GATA)$_{2}$TAT 3’	C (–23), T (+36)
**Du323(arm)3**	184	5’ (AC)$_{5}$...(GATA)(GGT)(GATA)$_{3}$(GAT)(GATA)TAT 3’	A (–23), C (+36)
Du323(mix)1	184	5’ (AC)$_{5}$…(GATA)(GGT)(GATA)$_{3}$GAT(GATA)TAT 3’	A (–23), C (+36)

Du323(val)1	220	5’ $(AC)_6$…$(GATA)_{15}$GAT 3’	C (–23), T (+36)
Du323(val)2	216	5’ $(AC)_6$…$(GATA)_{14}$GAT 3’	C (–23), T (+36)
Du323(val)3	211	5’ $(AC)_6$…$(GATA)_{10}$GAT$(GATA)_2$TAT 3’	C (–23), T (+36)
Du323(val)4	211	5’ $(AC)_6$…$(GATA)_5$(GACA)$(GATA)_4$GAT$(GATA)_2$TAT 3’	C (–23), T (+36)
Du323(val)5	164	5’ $(AC)_6$…$(GATA)_5$GAT$(GATA)_2$TAT 3’	C (–23), T (+36)
Du323(val)6	188	5’ $(AC)_6$…$(GATA)_4$GAT$(GATA)_2$TAT 3’	C (–23), T (+36)
**Du47G(arm)1**	188	5’ $(GATA)_{10}(GACA)_4(GATA)_2GAT(GATA)_2$ 3’	T (+7), A (+21), G (+52), T (+56)
**Du47G(arm)2**	184	5’ $(GATA)_9(GACA)_4(GATA)_2$GAT$(GATA)_2$ 3’	T (+7), A (+21), G (+52), T (+56)
**Du47G(arm)3**	176	5’ $(GATA)_{11}$(GACA)(GATA)GAT$(GATA)_2$ 3’	T (+7), T (+21), C (+52), A (+56)
**Du47G(arm)4**	172	5’ $(GATA)_{10}$(GACA)(GATA)GAT$(GATA)_2$ 3’	T (+7), T (+21), C (+52), A (+56)
**Du47G(arm)5**	168	5’ $(GATA)_9$(GACA)(GATA)GAT$(GATA)_2$ 3’	T (+7), T (+21), C (+52), A (+56)
**Du47G(arm)6**	164	5’ $(GATA)_8$(GACA)(GATA)GAT$(GATA)_2$ 3’	T (+7), T (+21), C (+52), A (+56)
**Du47G(arm)7**	152	5’ $(GATA)_2$(GACA)$(GATA)_4$GAT$(GATA)_2$ 3’	A (+7), A (+21), G (+52), A (+56)
Du47G(mix)1	152	5’ $(GATA)_2$(GACA)$(GATA)_4$GAT$(GATA)_2$ 3’	A (+7), A(+21), G (+52), A (+56)
Du47G(mix)2	148	5’ $(GATA)_2$(GACA)$(GATA)_3$GAT$(GATA)_2$ 3’	A (+7), A (+21), G (+52), A (+56)
Du47G(val)1	211	5’ $(GATA)_5$GAT$(GATA)_{14}$(GACA)(GATA)GAT$(GATA)_2$ 3’	T (+7), T (+21), C (+52), A (+56)
Du47G(val)2	200	5’ $(GATA)_{17}$(GACA)(GATA)GAT$(GATA)_2$ 3’	T (+7), T (+21), C (+52), A (+56)
Du47G(val)3	196	5’ $(GATA)_{16}$(GACA)(GATA)GAT$(GATA)_2$ 3’	T (+7), T (+21), C (+52), A (+56)
Du47G(val)4	192	5’ $(GATA)_{15}$(GACA)(GATA)GAT$(GATA)_2$ 3’	T (+7), T (+21), C (+52), A (+56)
Du47G(val)5	188	5’ $(GATA)_{14}$(GACA)(GATA)GAT$(GATA)_2$ 3’	T (+7), T (+21), C (+52), A (+56)
Du47G(val)6	184	5’ $(GATA)_{13}$(GACA)(GATA)GAT$(GATA)_2$ 3’	T (+7), T (+21), C (+52), A (+56)
Du47G(val)7	180	5’ $(GATA)_{12}$(GACA)(GATA)GAT$(GATA)_2$ 3’	T (+7), T (+21), C (+52), A (+56)
Du47G(val)8	176	5’ $(GATA)_{11}$(GACA)(GATA)GAT$(GATA)_2$ 3’	T (+7), T (+21), C (+52), A (+56)

Du47G(val)9	168	5’ $(GATA)_9$(GACA)(GATA)GAT$(GATA)_2$ 3’	T (+7), T (+21), C (+52), A (+56)
Du47G(val)10	164	5’ $(GATA)_8$(GACA)(GATA)GAT$(GATA)_2$ 3’	T (+7), T (+21), C (+52), A (+56)

Parental alleles are depicted.
