# Supplementary material for: Multiple interspecific hybridization and microsatellite mutations provide clonal diversity in the parthenogenetic rock lizard Darevskia armeniaca
Source: BMC Genomics. 2018 Dec 29;19:979. doi: 10.1186/s12864-018-5359-5 (PMC6311022; doi:10.1186/s12864-018-5359-5)
Supplement: Supplementary file 2 — Table S2. The population indices of gene diversity for four studied loci in twelve sampled populations of D. valentini. (PDF 99 kb) [file 12864_2018_5359_MOESM2_ESM.pdf]

Table S2 The population indices of gene diversity for four studied loci in twelve sampled populations of *D. valentini*

Locus	Population	Alelle (N)	R_S	H_E	H_O
Du215	Lchashen	1	1.00	-	0.00
	Kuchak	1	1.00	-	0.00
	Tezh	1	1.00	-	0.00
	Adis	1	1.00	-	0.00
	Total	1	1.00	-	0.00
	Mean ± SE	1 ± 0	1.00 ± 0.00	-	0 ± 0.00
Du281	Lchashen	3	1.79	0.6	0.80
	Kuchak	2	2.00	0.5	0.50
	Tezh	4	2.23	0.56	0.33
	Adis	2	2.24	0.43	0.50
	Total	5	5.00	0.56	0.53
	Mean ± SE	2.75 ± 0.48	2.06 ± 0.11	0.52 ± 0.04	0.53 ± 0.10
Du323	Lchashen	3	3.00	0.64	1.00
	Kuchak	3	3.00	0.83	0.50
	Tezh	2	2.3	0.55	1.00
	Adis	4	1.94	0.82	0.75
	Total	4	4.00	0.66	0.88
	Mean ± SE	3 ± 0.41	2.56 ± 0.26	0.71 ± 0.07	0.81 ± 0.12
Du47G	Lchashen	5	3.43	0.82	0.80
	Kuchak	3	4.00	0.83	1.00
	Tezh	6	2.96	0.82	1.00
	Adis	6	3.05	0.89	1.00
	Total	10	12.00	0.84	0.94
	Mean ± SE	5 ± 0.71	3.36 ± 0.24	0.84 ± 0.02	0.95 ± 0.05

N—number of alleles, R_S—allelic richness, H_E—expected heterozygosity, H_O—observed heterozygosity.
