# Supplementary material for: Multiple interspecific hybridization and microsatellite mutations provide clonal diversity in the parthenogenetic rock lizard Darevskia armeniaca
Source: BMC Genomics. 2018 Dec 29;19:979. doi: 10.1186/s12864-018-5359-5 (PMC6311022; doi:10.1186/s12864-018-5359-5)
Supplement: Supplementary file 3 — Table S3. Indices of association in four populations of D. valentini. (PDF 347 kb) [file 12864_2018_5359_MOESM3_ESM.pdf]

Table S3 Indices of association in four populations of *D. valentini*

Population	I_a	p	$\overline{r}_d$	p
Adis	-0.11	0.58	-0.06	0.609
Lchashen	0.85	0.03	0.45	0.009
Kuchak	-	-	-	-
Tezh	0.14	0.30	0.15	0.13
Total	0.01	0.48	0.002	0.47

I_a—index of association, $\overline{r}_d$—standardized index of association, p—p-value corresponding to index of association.
