# Supplementary figures and images for: Multiple interspecific hybridization and microsatellite mutations provide clonal diversity in the parthenogenetic rock lizard Darevskia armeniaca
Source: BMC Genomics. 2018 Dec 29;19:979. doi: 10.1186/s12864-018-5359-5 (PMC6311022; doi:10.1186/s12864-018-5359-5)

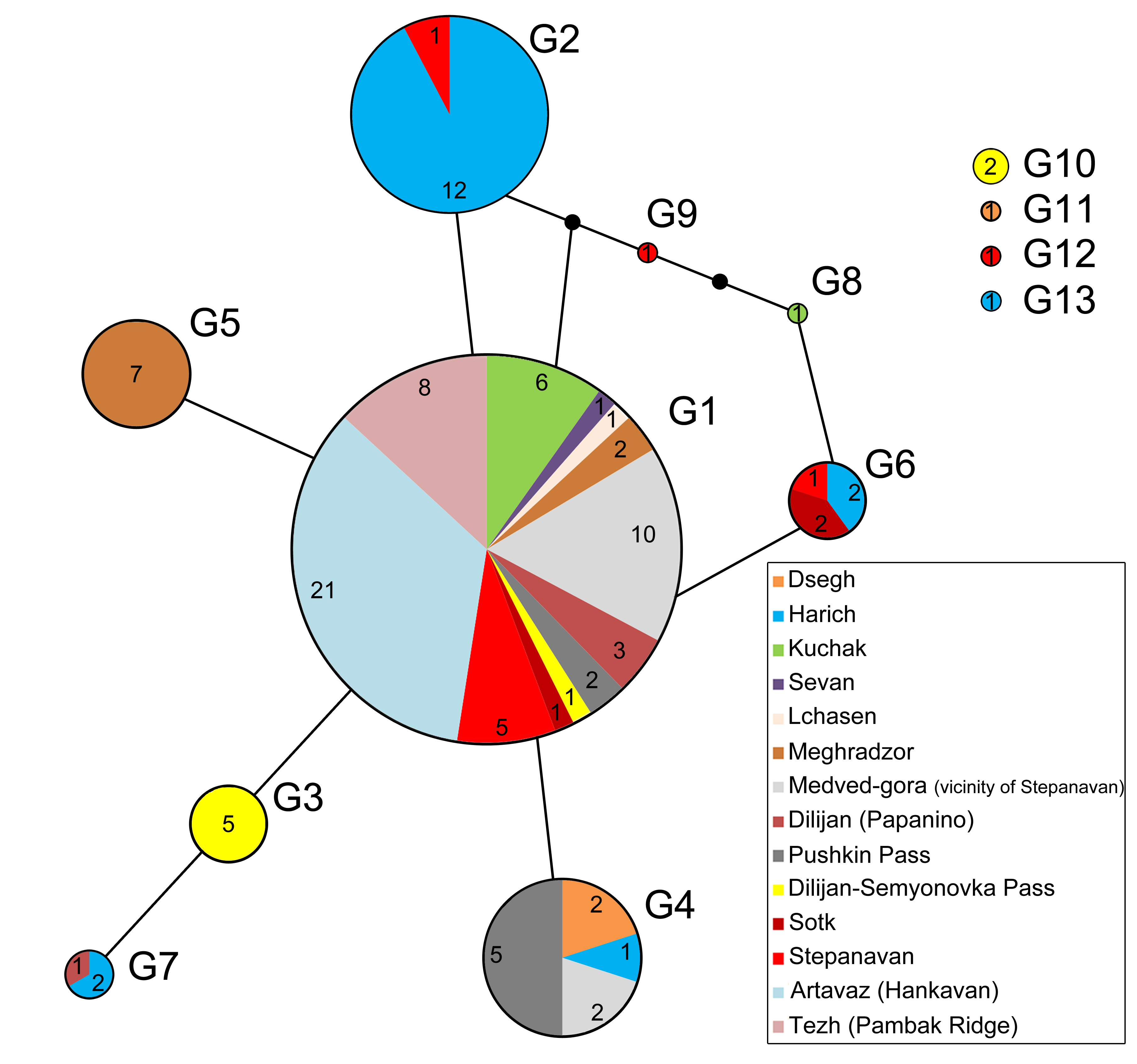

Supplement: Supplementary file 4 — Figure S1. Schematic representation of the TCS network that reflects distribution of genotypes 1–9 in D. armeniaca. Concatenated sequences of D. armeniaca genotypes were analyzed using TCS software v.1.21. Genotypes 10–13 are plotted separately. Population distribution of the genotypes is shown by different colors. Numbers indicate the number of individuals in populations. The black circles show, unsampled, but computer-predicted genotypes. (TIF 802 kb) [file 12864_2018_5359_MOESM4_ESM.tif]
